# Characteristics of the Side Surfaces and Edges of Welded Wire Meshes Used in the Construction of Welded Slotted Screens

**DOI:** 10.3390/ma16206701

**Published:** 2023-10-16

**Authors:** Mariusz Bąk, Sylwia Wencel, Paweł Wieczorek

**Affiliations:** 1 Progress Eco S.A., Dobrów 7, 28-142 Tuczępy, Poland; mbak@progress-screens.pl; 2 Faculty of Production Engineering and Materials Technology, Czestochowa University of Technology, Av. Armii Krajowej 19, 42-201 Czestochowa, Poland; pawel.wieczorek@pcz.pl

**Keywords:** slotted screens, duplex steel, wire surface

## Abstract

Welded resistance slotted screens, also known as slotted screens, are a special type of screen primarily used for the filtration and separation of liquids and dust. They are characterized by slots with parallel geometry and precisely defined sizes. The quality of the side surfaces and edges of welded wires determines the durability of the slotted screens made from them. This article presents the results of tests for four types of wires: two types of working profile wires made from austenitic-ferritic steel (duplex) and two types of supporting cross wires made from ferritic steel. The wire surfaces were characterized using a profilometer and atomic force microscopy. Basic roughness parameters Ra, Rz, and SAD (surface area difference) were determined. Surface observations of the working profiles were conducted using scanning electron microscopy. These studies allowed for the characterization of the working wire surfaces used in the production of slotted screens. At work, the results of surface roughness were analyzed based on three measurement methods for wires used in the production of welded slot screens. These results allowed for the identification of the most reliable method for characterizing the surface condition of such products.

## 1. Introduction

In recent years, we have witnessed significant advancements in the field of industrial metal production. Advanced manufacturing techniques such as powder metallurgy, selective laser melting (SLM), and computer-aided machining (CAM) have become widely adopted, enabling the precise production of metals with advanced properties [1,2,3,4]. However, that is not all—advanced numerical modeling and process optimization technologies allow for the improvement of manufacturing process parameters and waste minimization [5,6,7,8].

As the metal industry has evolved, progress has also been made in characterizing the mechanical properties and resistance of metal materials. Advanced testing techniques such as scanning electron microscopy (SEM), AFM microscopy, and non-destructive testing enable the precise examination of material structures and strength, which is crucial for ensuring the quality of manufactured products [9,10,11,12,13].

The design of the final product and its application in the industry are extremely important stages in the production process that impact the efficiency and success of industrial endeavors.

Product design allows for customization to specific industrial applications. Engineers can design a product to meet specific requirements and goals, influencing its functionality and utility in a particular industry.

Proper product design can significantly affect its performance and efficiency in the industry. By optimizing the design, materials, and production processes, better operational parameters and resource savings can be achieved.

Product design takes into account durability and reliability. A well-designed product is more likely to operate without failures for an extended period, which is crucial in an industry where downtime can be costly [14,15,16,17].

**Figure 1:**
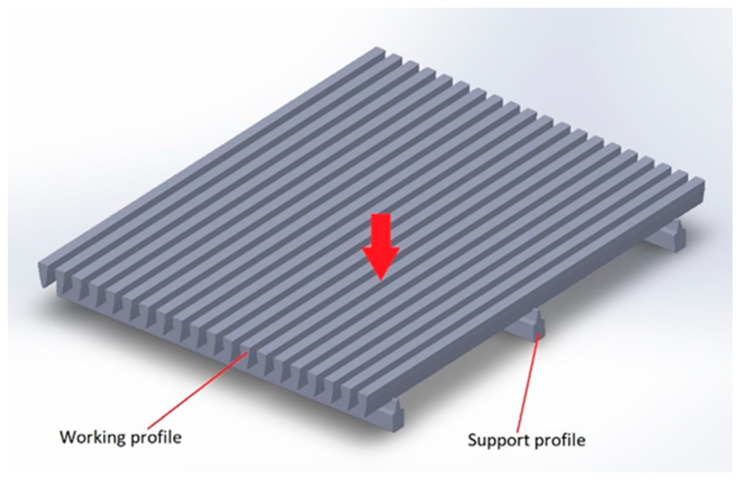
Slotted screen.

Welded slotted screens [18], as shown in Figure 1, represent some of the most technologically advanced slotted screen designs. The technology of resistance welding of specially profiled working wires to support wires allows for the attainment of highly precise slot dimensions, which play a crucial role in numerous solid–liquid filtration processes [19,20,21].

Welded slotted screens are characterized by the following:

- The ability to withstand heavy loads;
- A high open surface area coefficient;
- Low susceptibility to clogging;
- Even and smooth surfaces of the working wires;
- High precision in manufacturing;
- High efficiency and accuracy in separation and dewatering processes.

To achieve the aforementioned properties, slotted screens must be made from the working profile wires of corrosion-resistant steel (austenitic, ferritic, duplex austenitic-ferritic). The production process of the working profiles and support wires must ensure appropriate strength parameters, minimal roughness of the working and side surfaces, proper filling of the profiles and support wires during plastic processing, and high dimensional accuracy (narrow tolerance range).

The quality of welded surfaces, as determined via surface finish, contaminants, and surface defects, clearly affects the strength of the welded joint. The improper preparation of welded surfaces can result in the presence of post-welding microcracks, porosity, and delamination, arising not only from the welding process itself but also from the presence of post-process surface contaminants [22,23].

To meet the quality requirements of working wires and support wires by ensuring the necessary precision of these components, it is essential to appropriately design the metal forming process during plastic deformation. The primary processes in which this can be achieved are drawing processes [24] or wire rolling [25].

Due to the need to maintain dimensions, the drawing process is applied sporadically and mostly for support wires, as the dimensions of the wires change due to die wear [26,27,28].

In the case of wire rolling, the wire profile is created by flattening the wire and rolling it until the final shape is achieved using so-called “Turkish cages” [29]. The greatest advantage of cold rolling is the ability to produce a product with precise dimensions and excellent surface quality. The surface is smooth, free of scale, shiny, and without scratches or stains. The material is cold-rolled, which prevents recrystallization processes that would negatively affect the physical properties of the resulting product. Work hardening increases the hardness and tensile strength of the produced profiles, resulting in high operational properties. Their load-bearing capacity increases, as does fatigue strength and wear resistance. Among the other requirements placed on materials for slotted screens are corrosion resistance and high surface quality [30]. The surface roughness and corrosion resistance of these semi-finished products are crucial during the production of slotted screens.

Reducing the surface roughness of working and support wires leads to an increase in the operational life of slotted screens. The lifespan of slotted screens depends on wire strength, dimensional tolerances, and residual stresses, which can accumulate during metal forming and spot welding [31,32,33].

One promising group of materials for slotted screens is duplex stainless steel. The advantages of this steel group over austenitic and ferritic corrosion-resistant steels include up to twice the tensile strength, high ductility, excellent corrosion resistance, and economic factors, as duplex steels contain lower levels of molybdenum and nickel, making them cheaper to produce in the mill than many traditional austenitic steels. The price of duplex steel is also more stable than other types of steel, making cost estimation easier [34].

Higher strength and corrosion resistance also mean that many components made using duplex stainless steel can be thinner than their austenitic counterparts.

The second group of steels analyzed in this study is ferritic stainless steels, which have good mechanical properties. Ferritic steels are characterized by a high yield strength, higher machinability, good thermal conductivity, and greater stress corrosion resistance induced by chlorides than austenitic steels. Ferritic steels contain a minimum of 10.5% chromium with minimal nickel content with other elements, including molybdenum, aluminum, and titanium. Ferritic steels exhibit high resistance to plastic deformation when stabilized with niobium.

The absence of expensive nickel in ferritic steel translates into an attractive price compared to austenitic steel. Thanks to these factors, these steels find wide application in industry.

This article presents the results of the surface quality analysis of wires used for support and working wires to assess their suitability for use in the slotted screen welding process.

The research was conducted using roughness measurements performed with a profilometer and an atomic force microscope (AFM). By utilizing the atomic force microscope, a linear analysis of surface roughness values and a surface analysis were carried out. The conducted studies allowed for the identification of the most reliable method for characterizing the surface condition of wires used in the production of welded slot screens.

## 2. Material Used for Research and Research Methodology

In this study, the profiles of working wires 34Sbb and 42Sbb, as well as support wires 34Sb and Q55, were subjected to analysis. These profiles were made from two types of corrosion-resistant steel: the working wires labeled 34Sbb and 42Sbb were made from duplex steel grade 1.4482, while the support wires 34Sb and Q55 had a ferritic structure and were made from steel grade 1.4016. The selection of these material combinations was influenced by an analysis of the suitability of duplex and ferritic steel in a mildly corrosive environment. The primary material selection criteria for the working and support wires were wear resistance, weldability, and economic aspects.

The aim of this research was to provide a comprehensive characterization of the condition of the side surfaces and edges of the welded analyzed working and support wires after the profile rolling process.

The wires were manufactured on a rolling line consisting of an uncoiler, rolling cages, a cleaning system, and a winder. In the first cage of the so-called duo rolling mill, round wires underwent a flattening process, and in the subsequent two non-driven so-called “Turkish” cages, the wires were rolled to achieve their final shape.

**Figure 2:**
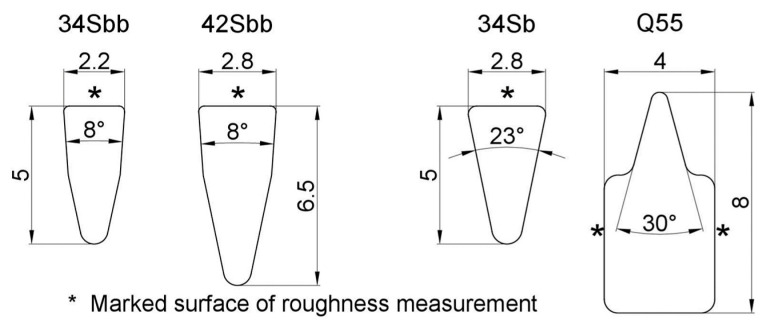
Wire profiles and dimensions obtained after rolling process.

The shape and dimensions of the profiles obtained in the process are presented in Figure 2.

In this study, the chemical composition of the steel grades used in the production of support and working wires was analyzed. The analysis was conducted using an Olympus Vanta XRF spectrometer. The results of the analysis are presented in Table 1.

The surface roughness assessment was conducted on the areas marked with an asterisk in Figure 2.

A MarSurf PS 10 profilometer was used for the surface roughness assessment.

For evaluating the quality of the welded edges of profiled wires, a JEOL 6610 scanning microscope was utilized. The scanning microscope was also employed to assess the surface condition of the areas indicated by an asterisk in Figure 2.

Atomic force microscopy with a VEECO Multimode 8 was also employed for surface roughness assessment. The examinations were conducted in two directions, namely in the rolling direction and perpendicular to the rolling direction.

## 3. Research Results

The analysis of the condition of the side surfaces and welded edges using scanning microscopy was carried out for all wire and profile types.

**Figure 3:**
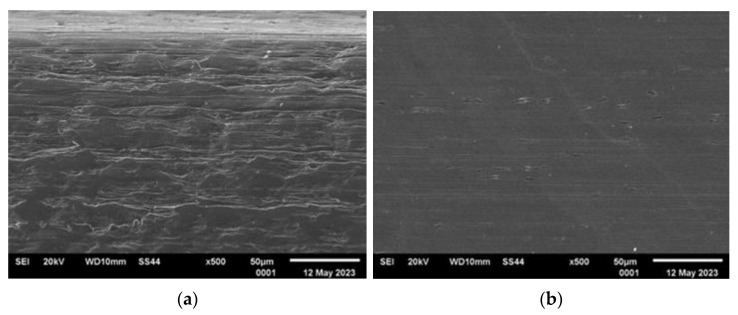
Condition of analyzed surfaces of working wire 34Sbb after rolling process: (**a**) profile edge (welded surface) and (**b**) working surface marked with “*” in Figure 2.

**Figure 4:**
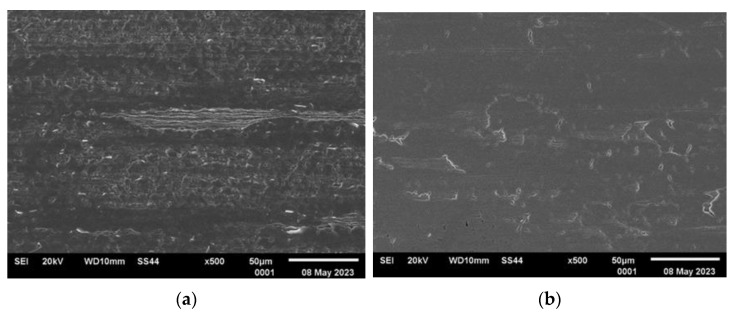
Condition of analyzed surfaces of working wire 42Sbb after rolling process: (**a**) profile edge (welded surface) and (**b**) working surface marked with “*” in Figure 2.

**Figure 5:**
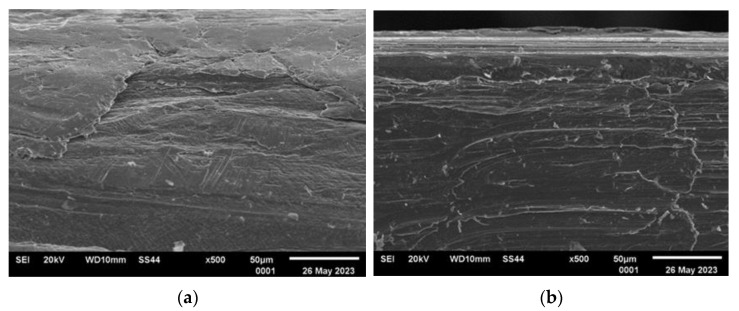
Condition of analyzed surfaces of supporting wire 34Sb after rolling process: (**a**) profile edge (welded surface) and (**b**) working surface marked with “*” in Figure 2.

**Figure 6:**
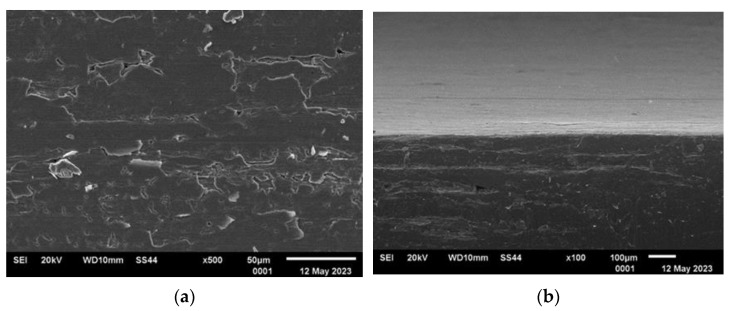
Condition of analyzed surfaces of supporting wire Q55 after rolling process: (**a**) profile edge (welded surface) and (**b**) working surface marked with “*” in Figure 2.

Figure 3, Figure 4, Figure 5 and Figure 6 present the representative images of the working surfaces and edges subject to welding in subsequent technological processes of manufacturing slotted screens.

The surface condition examinations revealed that profiles labeled 34Sbb and 42Sbb, made from duplex steel, exhibited a significantly smooth surface with few tears. The edges were continuous, sharp, and retained their specified shape along the entire length. This is crucial, especially for working wires, due to the required slot precision and working conditions. On the edge of the 42Sbb profile, defects resulting from material flaking during rolling were visible. Observations of the 34Sb profile indicated the presence of defects both on the welded edge in the form of rolled-in material and on the working surface. Higher surface development with traces mirroring the condition of the rolling tool surface could be observed.

The analysis of the examined surfaces in the case of the Q55 profile indicated that there were not as significant defects in the edge area as in the case of the 34Sb profile. However, the side surfaces marked in Figure 2 featured a series of tears and scratches related to the low material plasticity and the condition of the rolling tool surface.

Generally, surface defects visible on the welding edges were caused by the free flow of material and were largely associated with the material’s plasticity. These defects were more numerous than on flat surfaces where the profile comes into contact with the forming tool.

**Figure 7:**
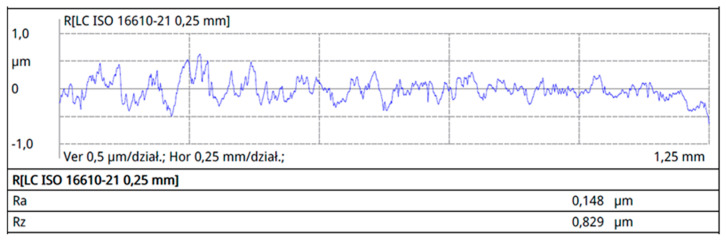
Roughness profile of the working profile 34Sbb on the front working surface.

**Figure 8:**
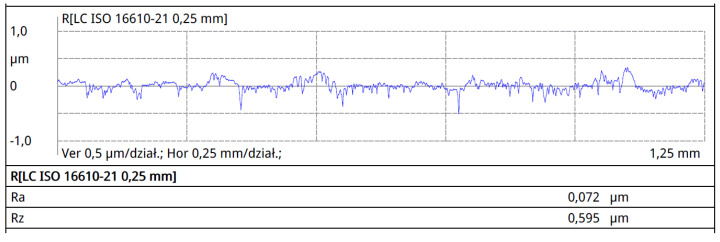
Roughness profile of the working profile 42Sbb on the front working surface.

**Figure 9:**
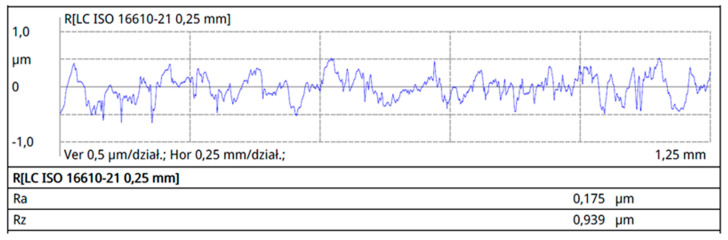
Roughness profile of the support wire (crosspiece) 34Sb on the lateral surface.

**Figure 10:**
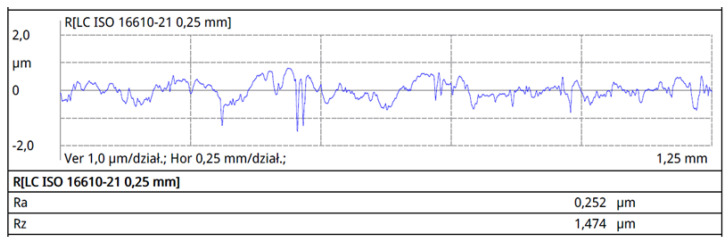
Roughness profile of the support wire (crosspiece) Q55 on the lateral surface.

Surface roughness tests were conducted on the surfaces marked with an asterisk in Figure 2, perpendicular to the rolling direction. The results of surface roughness measurements for the individual profiles are presented in Figure 7, Figure 8, Figure 9 and Figure 10.

The collective compilation of roughness parameters obtained from the profilometer measurements is presented in Table 2.

Analyzing the roughness measurement results, it can be observed that the roughness values for working wires are lower than those for support wires. Wire roughness affects the efficiency and durability of screens; therefore, for the profiles of working wires, a roughness value of Ra below 0.3 μm is expected. The surface quality of the tools and the material being processed significantly influence roughness. The production tools for profiles are standardized in terms of roughness, and for standard tools, a maximum Ra value of 0.15 μm has been specified. Despite using tools for producing 34Sbb and 34Sb with similar roughness, the Ra parameter for the 34Sbb profile was 0.171 μm, while for the support wire 34Sb, it was higher at 0.218 μm. This difference is due to the properties of the material from which the details are made. Profiles made from steel grade 1.4482 have a plastic austenitic structure and, as a result, exhibit lower roughness after the rolling process.

The lowest Ra roughness value was recorded for the 42Sbb profile made from steel grade 1.4482, and it was 0.074 μm. The highest level of roughness for the Ra parameter was observed for the Q55 profile of the support wire, reaching 0.502 μm. This parameter was not critical for this type of wire, as it is a support wire where a very low roughness value is not required.

**Figure 11:**
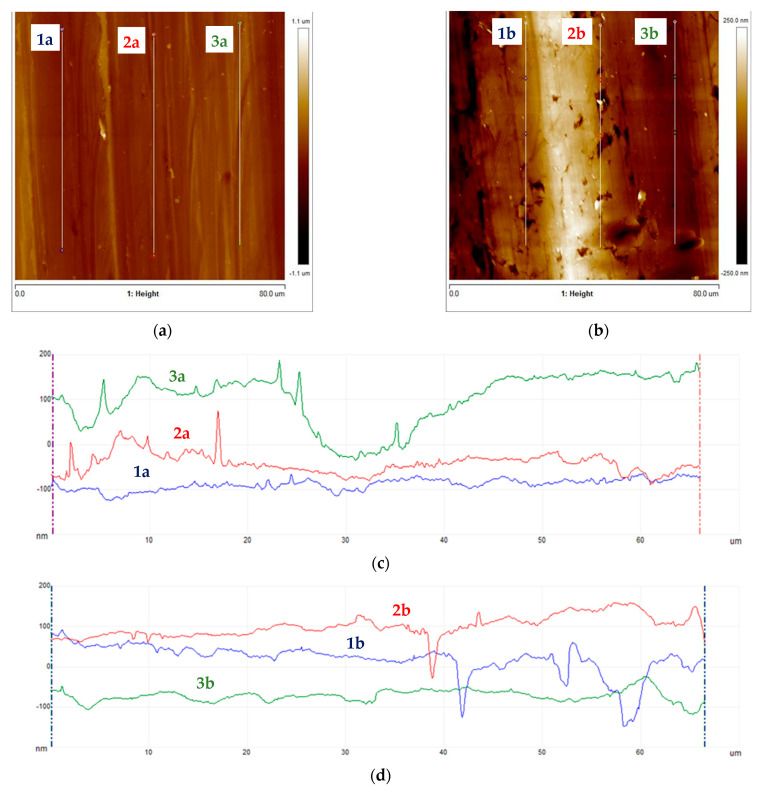
Working wires topography: (**a**) 34Sbb profile with marked roughness measurement lines, (**b**) 42Sbb profile with marked roughness measurement lines, (**c**) roughness profiles for 34Sbb measured parallel to the rolling direction, and (**d**) roughness profiles for 42Sbb measured parallel to the rolling direction.

**Figure 12:**
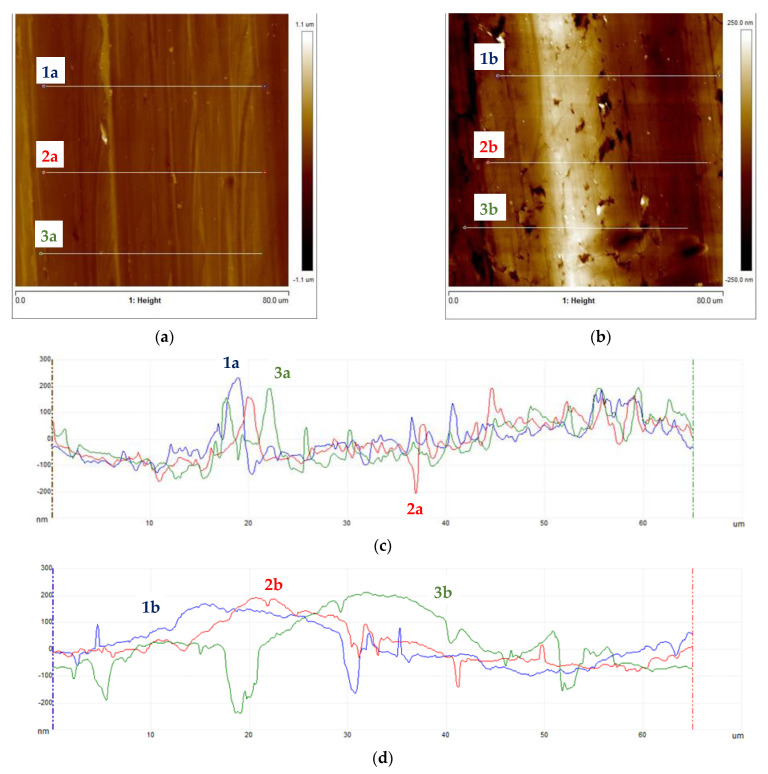
Working wires topography: (**a**) 34Sbb profile with marked roughness measurement lines, (**b**) 42Sbb profile with marked roughness measurement lines, (**c**) roughness profiles for 34Sbb measured perpendicular to the rolling direction, and (**d**) roughness profiles for 42Sbb measured perpendicular to the rolling direction.

**Figure 13:**
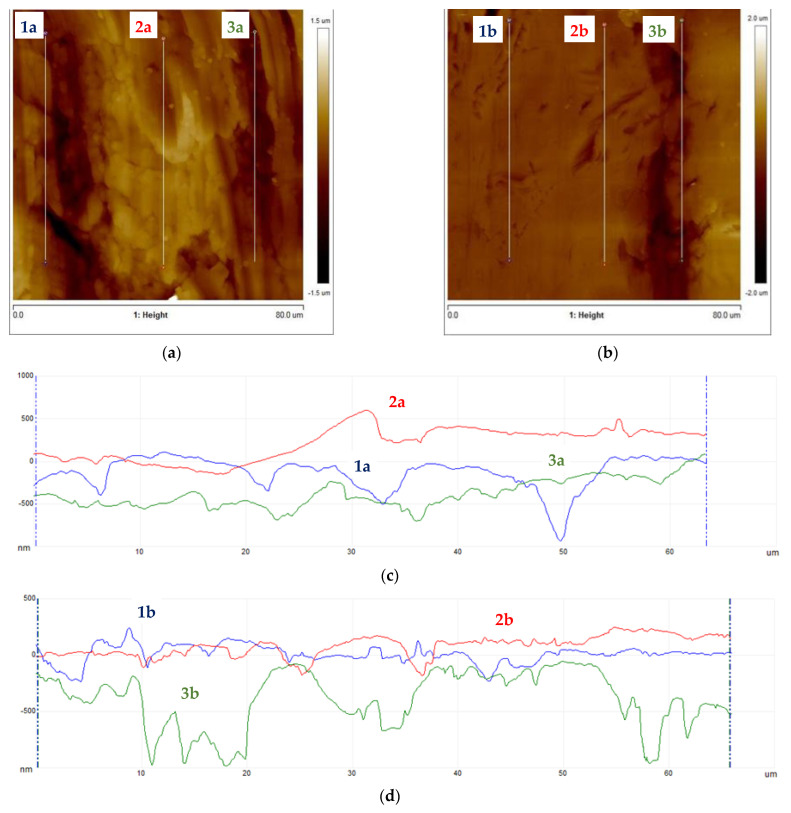
Support wires topography: (**a**) 34Sb with marked roughness measurement lines, (**b**) Q55 with marked roughness measurement lines, (**c**) roughness profiles for 34Sb measured parallel to the rolling direction, and (**d**) roughness profiles for Q55 measured parallel to the rolling direction.

**Figure 14:**
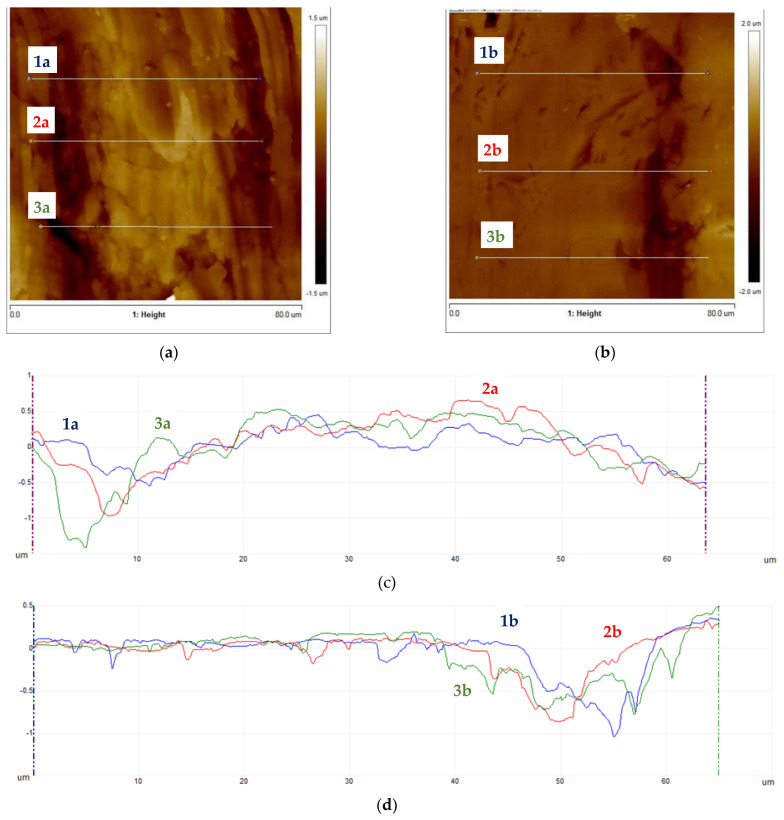
Support wires topography: (**a**) 34Sb with marked roughness measurement lines, (**b**) Q55 with marked roughness measurement lines, (**c**) roughness profiles for 34Sb measured parallel to the rolling direction, and (**d**) roughness profiles for Q55 measured parallel to the rolling direction.

Subsequently, samples were subjected to micro-area roughness measurements marked with an asterisk in Figure 2 using a Brucker Multimode 8 atomic force microscope. The results of the measurements are presented in Figure 11, Figure 12, Figure 13 and Figure 14 and in Table 3.

Working profile wires made from duplex steel had similar roughness values for measurements perpendicular to the rolling direction and smaller but similar values for measurements parallel to the rolling direction. The roughness measurement results are significantly higher for AFM measurements than those obtained using a profilometer. This is a consequence of the higher precision of scanning the surfaces under examination when using an atomic force microscope. Measurements made with a profilometer are conducted over a longer measurement length and are more representative of production analysis. For the profiles of working wires, a roughness of Ra below 0.3 μm is expected and required for both rolling directions. Significant differences in roughness values for profiles made from duplex steel depending on the rolling direction are observed, with Ra values of 0.024 μm and 0.019 μm (Table 3) for measurements lengthwise to the rolling direction. Meanwhile, for measurements perpendicular to the rolling direction, these values are 0.118 μm and 0.068 μm, respectively. However, differences in roughness values depending on the rolling direction are smaller for support wires made from ferritic steel.

**Figure 15:**
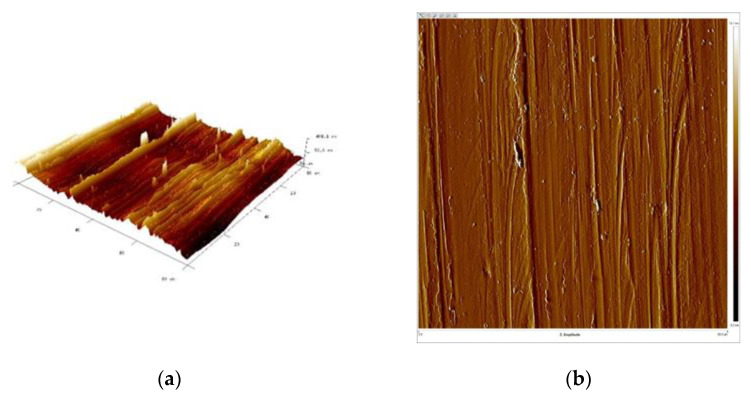
Working wire 34Sbb: (**a**) 3D surface view and (**b**) surface amplitude contrast.

**Figure 16:**
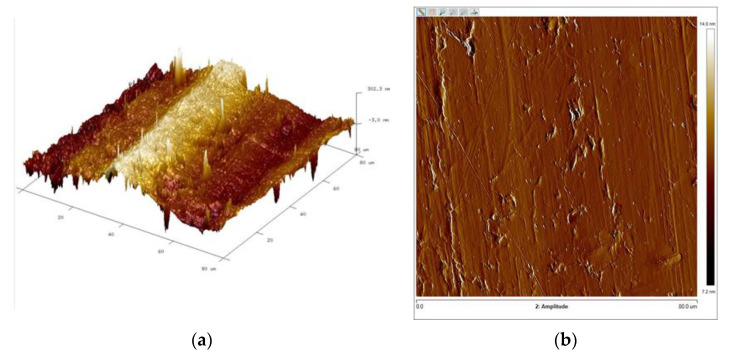
Working wire 42Sbb: (**a**) 3D surface view and (**b**) surface amplitude contrast.

**Figure 17:**
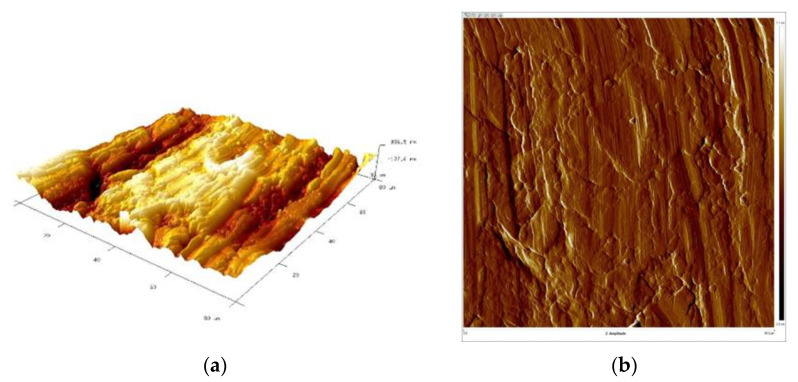
Support wire 34Sb: (**a**) 3D surface view and (**b**) surface amplitude contrast.

**Figure 18:**
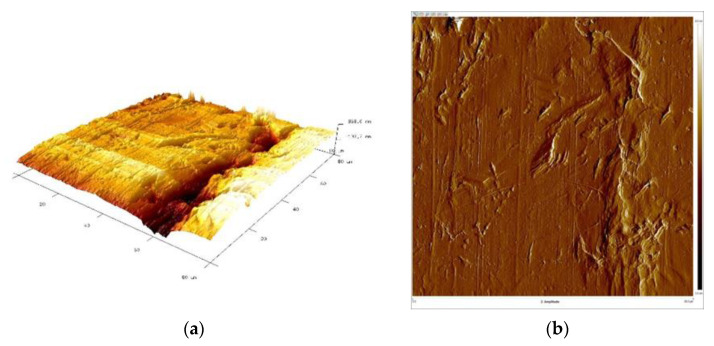
Support wire Q55: (**a**) 3D surface view and (**b**) surface amplitude contrast.

Figure 15, Figure 16, Figure 17 and Figure 18 present 3D views of the details’ surfaces along with the surface roughness results. An extremely important parameter for assessing the degree of surface development of the details is SAD, defined as the ratio of the actual surface area to the projection area of the research area.

The roughness parameters determined from the 3D images in Figure 15 were as follows: SAD-0.90%, Rq-0.132 μm, and Ra-0.108 μm; SAD-0.74%, Rq-0.111 μm, and Ra-0.077 μm in Figure 16; SAD-2.11%, Rq-0.308 μm, and Ra-0.247 μm in Figure 17; and SAD-1.94%, Rq-0.182 μm, and Ra-0.120 μm in Figure 16.

The support wire 34Sb had the most developed surface with an SAD of approximately 2.11% (Figure 17). Profiles 34Sbb and 42Sbb made from 1.4482-grade steel had similar values for Ra, Rz, and SAD (Figure 15 and Figure 17). Profile 42Sbb, made from duplex steel, had the smallest values for Ra, Rz, and SAD among all the tested samples. Support wires 34Sb and Q55 made from 1.4016-grade steel had higher values for Ra, Rz, and SAD compared to profiles made from duplex steel.

## 4. Discussion of Research Results

In this article, the topography of the working surfaces of support wires and working profiles used in the production of slotted screens was characterized.

The 34Sbb profile made of 1.4482-grade steel had a smooth surface with few interruptions. The Ra measured via the profilometer was 0.171 μm. Max indentations of 0.150 μm were identified for measurements along the rolling direction, and max elevations of 0.200 μm were identified for measurements perpendicular to the rolling direction. The SAD was 0.90%.

The 42Sbb working profile made of 1.4482-grade steel had a smooth surface with few interruptions. The Ra measured via the profilometer was 0.074 μm, and occasional scratches with a depth of 0.100 μm were observed for measurements along the rolling direction. Indentations and elevations of 0.200 μm were also observed. For measurements perpendicular to the rolling direction, single scratches with a depth of 0.150 μm were observed. The SAD was 0.74%.

Low surface roughness values in the case of working wires are largely associated with the structural composition of duplex steel. The presence of austenite in the duplex steel structure limits the peeling effect of the surface due to the interaction with the shaping tool pressure (rollers) during the profile rolling process.

The support wire 34Sb made of 1.4016-grade steel exhibited material tearing and marks that reflected the tool on the surface. The Ra measured via profilometer was 0.218 μm, and maximum indentations and elevations of 0.500 μm, as well as scratches of size 1.000 μm, were identified for measurements along the rolling direction. For measurements perpendicular to the rolling direction, indentations of 1.000 μm in depth and elevations of 0.500 μm were identified. The SAD was 2.11%.

The support wire Q55, made of 1.4016-grade steel, had a smooth surface with few interruptions. The Ra measured via the profilometer was 0.502 μm, and significant indentations/scratches of 1.000 μm were observed for measurements along the rolling direction. For measurements perpendicular to the rolling direction, indentations of 1.000 μm and scratches of 0.200 μm were observed. The SAD was 1.94%.

The structural composition of support wires, primarily consisting of ferrite, exhibits lower ductility compared to the structure of profile wires. During the profile shaping of support wires in the rolling process, there is the surface strengthening of the wires, leading to the generation of microcracks and resulting in the occurrence of the pitting phenomenon (Figure 6, Figure 17 and Figure 18).

The roughness parameters of both working and support profiles were determined using the quality of the tool surface and the properties of the processed material.

The level of surface roughness obtained for the working wires directly affects the operational properties of the slotted screens, regardless of the analyzed area. The lower the roughness level of the working wire surfaces, the higher the operational properties of the slotted screens made from them.

Therefore, this article analyzed the level of side surface roughness of the working wires, both parallel and perpendicular to the rolling direction.

It was found that there was a significantly higher roughness value in the case of measurements made perpendicular to the rolling direction.

Working wires after the rolling process, regardless of the direction of the analyzed process, achieved a required roughness level of 0.15 μm.

The best roughness value was obtained for the working wire of profile 42Sbb.

In addition, the roughness values for these profiles were characterized by the lowest variability of results.

However, the roughness values for support wires of profiles 34Sb and Q55 were significantly higher than for working wires despite using the same quality tools in the rolling process.

The increase in roughness level was due to the type of steel used to make the specific wire and its susceptibility to plastic deformation.

A comprehensive view of the surface development degree in rolled wires is provided by the SAD (surface area density) results obtained from the analysis of the examined wire surfaces. In the case of working wires, the result is below 1% (0.9% for 34Sbb and 0.74 for 42Sbb), indicating a significantly lower surface development degree and, consequently, a smoother surface. SAD for support wires was determined at a level close to 2% (2.11% for 34SB and 1.94% for Q55). Unlike the conducted linear analysis, SAD encompasses surface analyses and characterizes the analyzed surfaces more precisely in terms of surface stereology.

## 5. Conclusions

The surface roughness level investigations conducted in this study for support and working wires have allowed for the formulation of the following conclusions:

1. The analysis of microscopic examination results using scanning microscopy for working wires and support wires for areas that will undergo the welding process allows for the selection of profile 42Sbb for working wire and profile Q55 for support wire as optimal in terms of surface condition obtained after rolling.

2. The conducted research on the level of surface roughness, regardless of the type of wire and profile analyzed, indicates a significantly higher accuracy of the research method used to assess roughness using atomic force microscopy.

3. The results of the surface roughness level obtained using atomic force microscopy are several times lower than those obtained using a profilometer. Due to the significant influence of wire roughness levels used in the production of slotted screens, it is recommended to use atomic force microscopy to assess this parameter.

4. The conducted 3D analysis using an atomic force microscope allowed for the determination of stereological parameters independently of the roughness measurement direction, as was the case with profilometer measurements or linear analysis using AFM. The calculated SAD parameter enabled a comprehensive assessment of surface development for the analyzed wires.

5. The analysis of surface roughness levels for working wires made of duplex-type steel, regardless of the profile type, allows for the conclusion that the rolling process conducted enabled obtaining material that meets the quality requirements regarding the roughness of profiled working wires used on slotted screens, which must not exceed 0.15 μm.

6. As a result of the conducted research on the surface roughness of working wires and support wires, the optimal profile variants for the welding process were selected. It is recommended to use profile 42Sbb for working wires and profile 34Sb for support wires.

## Figures and Tables

**Table 1:** Chemical composition of analyzed steel grades, wt%.

Profile	Steel Grade	Chemical Composition, %								
		C	Si	Mn	S	P	Cr	Ni	Mo	N
34Sbb	1.4482	0.028	0.55	4.135	0.011	0.017	20.21	1.80	0.436	-
42Sbb	1.4482	0.019	0.65	4.115	0.001	0.026	20.15	1.79	0.449	-
34Sb	1.4016	0.020	0.43	0.4	0.011	0.037	16.04	0.50	-	0.035
Q55	1.4016	0.028	0.40	0.53	0.014	0.029	16.16	0.46	-	0.030

**Table 2:** Results of roughness level measurements for working wires and support wires determined using a profilometer.

Working Wire 34Sbb	Perpendicular to the Rolling Direction	
	Ra [μm]	Rz [μm]
mean value	0.171	0.899
standard deviation	0.087	0.464
Working wire 42Sbb	perpendicular to the rolling direction	
	Ra [μm]	Rz [μm]
mean value	0.074	0.599
standard deviation	0.037	0.300
Supporting wire 34Sb	perpendicular to the rolling direction	
	Ra [μm]	Rz [μm]
mean value	0.218	1.308
standard deviation	0.113	0.714
Supporting wire Q55	perpendicular to the rolling direction	
	Ra [μm]	Rz [μm]
mean value	0.502	2.833
standard deviation	0.258	1.465

**Table 3:** Results of roughness level measurements for working wires and support wires determined using an atomic force microscope.

Working Wire 34Sbb	Perpendicular to the Rolling Direction		Lengthwise to the Rolling Direction	
	Ra [μm]	Rz [μm]	Ra [μm]	Rz [μm]
mean value	0.118	0.291	0.024	0.072
standard deviation	0.085	0.148	0.014	0.033
Working wire 42Sbb	perpendicular to the rolling direction		lengthwise to the rolling direction	
	Ra [μm]	Rz [μm]	Ra [μm]	Rz [μm]
mean value	0.068	0.122	0.019	0.041
standard deviation	0.011	0.034	0.007	0.017
Supporting wires 34Sb	perpendicular to the rolling direction		lengthwise to the rolling direction	
	Ra [μm]	Rz [μm]	Ra [μm]	Rz [μm]
mean value	0.269	0.705	0.181	0.499
standard deviation	0.066	0.139	0.082	0.210
Supporting wire Q55	perpendicular to the rolling direction		lengthwise to the rolling direction	
	Ra [μm]	Rz [μm]	Ra [μm]	Rz [μm]
mean value	0.114	0.165	0.073	0.199
standard deviation	0.065	0.083	0.070	0.268

## Data Availability

Data sharing is not applicable to this article.
